# Two Classes of T1 Hypointense Lesions in Multiple Sclerosis With Different Clinical Relevance

**DOI:** 10.3389/fneur.2021.619135

**Published:** 2021-03-03

**Authors:** Krisztián Kocsis, Nikoletta Szabó, Eszter Tóth, András Király, Péter Faragó, Bálint Kincses, Dániel Veréb, Bence Bozsik, Katalin Boross, Melinda Katona, Péter Bodnár, Nyúl Gábor László, László Vécsei, Péter Klivényi, Krisztina Bencsik, Zsigmond Tamás Kincses

**Affiliations:** ^1^Department of Neurology, Albert Szent-Györgyi Clinical Center, University of Szeged, Szeged, Hungary; ^2^Department of Radiology, Albert Szent-Györgyi Clinical Center, University of Szeged, Szeged, Hungary; ^3^Department of Image Processing and Computer Graphics, University of Szeged, Szeged, Hungary; ^4^Magyar Tudományos Akadémia-Szegedi Tudományegyetem (MTA-SZTE) Neuroscience Research Group, Szeged, Hungary

**Keywords:** multiple sclerosis, T1 hypointense lesions, black holes, MRI protocol, clustering, clinical state

## Abstract

**Background:** Hypointense lesions on T1-weighted images have important clinical relevance in multiple sclerosis patients. Traditionally, spin-echo (SE) sequences are used to assess these lesions (termed black holes), but Fast Spoiled Gradient-Echo (FSPGR) sequences provide an excellent alternative.

**Objective:** To determine whether the contrast difference between T1 hypointense lesions and the surrounding normal white matter is similar on the two sequences, whether different lesion types could be identified, and whether the clinical relevance of these lesions types are different.

**Methods:** Seventy-nine multiple sclerosis patients' lesions were manually segmented, then registered to T1 sequences. Median intensity values of lesions were identified on all sequences, then K-means clustering was applied to assess whether distinct clusters of lesions can be defined based on intensity values on SE, FSPGR, and FLAIR sequences. The standardized intensity of the lesions in each cluster was compared to the intensity of the normal appearing white matter in order to see if lesions stand out from the white matter on a given sequence.

**Results:** 100% of lesions on FSPGR images and 69% on SE sequence in cluster #1 exceeded a standardized lesion distance of *Z* = 2.3 (*p* < 0.05). In cluster #2, 78.7% of lesions on FSPGR and only 17.7% of lesions on SE sequence were above this cutoff value, meaning that these lesions were not easily seen on SE images. Lesion count in the second cluster (lesions less identifiable on SE) significantly correlated with the Expanded Disability Status Scale (EDSS) (*R*: 0.30, *p* ≤ 0.006) and with disease duration (*R*: 0.33, *p* ≤ 0.002).

**Conclusion:** We showed that black holes can be separated into two distinct clusters based on their intensity values on various sequences, only one of which is related to clinical parameters. This emphasizes the joint role of FSPGR and SE sequences in the monitoring of MS patients and provides insight into the role of black holes in MS.

## Introduction

Multiple sclerosis (MS) is a chronic, progressive disease affecting the central nervous system in young adults leading to demyelination and axonal loss (1). Conventional T1 (T1w)-and T2-weighted (T2w) magnetic resonance (MR) imaging has a key role in the diagnosis and in the follow-up of MS, but the relationship between clinical symptoms and white matter lesions is not straightforward (2). White matter lesions on T2w images are hyperintense and correspond to several histopathological changes such as edema or demyelination (3).

In contrast, hypointense white matter lesion on T1w images, the so-called black holes (BHs) correspond to axonal loss (4). The first description of the association between BHs and the clinical state of MS patients was published by Truyen et al. Using spin-echo (SE) MRI sequences in their 40-month follow-up study they showed that patients' baseline disabilities correlated significantly with BH lesion load, and in the case of secondary progressive MS, the accumulation of BHs contributed to the progression rate as well. They suggested that the presence of BHs led to a failed remission (5). BHs could be divided into two major types, namely, acute and persisting BHs. Acute BHs are contrast-enhancing lesions, and during the course of the disease, 20–40% of the acute BHs turns into persisting BHs (4, 6). BHs have high water content and correspond to irreversible MS pathology (7–10). In addition, the pattern of spatial distribution of BHs has preferred loci in the brain; for example, BHs are rarely present around the lateral ventricles but appear more often in the supratentorial region, with an effect on supratentorial brain volume and consequently cerebral atrophy (11). It was also reported that BHs may play a role in the conversion of clinically isolated syndrome to MS (12).

Many studies investigated the possible contribution of BHs to the clinical and cognitive state or their worsening in patients with MS. Based on these studies, BHs may present a possible resolution to the clinico-radiological paradox. Cognitive dysfunctions, reported in 43–70% of MS patients, mainly affect the information processing speed, visuospatial memory, and executive functions (13, 14). Parietal and frontal BHs were shown as a significant predictor of attention and non-verbal intelligence as well as phonemic verbal fluency deficit (7). Giorgio et al. demonstrated in a follow-up study that BH lesion load and lesion count were associated not just with the baseline EDSS scores (which quantifies and monitors the disability of MS patients), but also with its worsening over a 10-year period (8).

After it was established that BHs contribute to the course and progression of the disease and have clinical relevance in MS, the main focus of researches was directed toward whether any variation in BH appearance could have clinical relevance. There are only a few studies conducted in this regard. Adusumilli et al. showed that BHs and gray holes (e.g., less hypointense lesions), presumably measured on SE images can be differentiated with high reliability and correlated well with different clinical and cognitive measures (15). Tam in another study proved that BHs with lower intensity compared to other BHs have higher clinical relevance (16).

Importantly, the correlation between T1 hypointense lesions to pathological features and clinical symptoms was mainly investigated on SE T1 weighted images. The downside of this acquisition technique is the relatively longer acquisition time. Fast spoiled gradient-echo (FSPGR) sequences are suitable alternatives providing shorter acquisition times and/or improved resolution. Hypointense lesions are also detectable on FSPGR sequences, but the clinical relevance and the possible differences in comparison to SE sequences have not been thoroughly studied.

In this study, we aimed (i) to determine whether the contrast difference between white matter hypointensities and the surrounding normal white matter is similar on SE and FSPGR images, (ii) if two different lesion types could be identified based on the intensities on the two sequences, and (iii) if the clinical relevance of the two lesion types are different.

## Methods

### Patients

In this study, 79 patients with relapsing-remitting MS (RRMS) diagnosis were enrolled. Patients were recruited from the Multiple Sclerosis Outpatient Clinic at the Department of Neurology, University of Szeged. Clinical and demographic parameters of the patient population are depicted in Table 1. All patients were in stable clinical state, no relapses occurred 6 months preceding and following the study. This study was approved by the Ethical Committee of the University of Szeged (Ref.No.: 000002/2016/OTIG). All study participants gave their written informed contribution in accordance with the Declaration of Helsinki.

**Table 1:** Demographic and clinical data of the patients.

**Number of** **patients**	**Females**	**Age (years)** **(mean ± std)**	**EDSS** **(median ± range)**	**Disease duration (years) (mean ± std)**	**Number of relapses since treatment onset; median (range)**	**Treatment regimen**	**Lesion load (mm^**3**^)** **(mean ± std)**
79	55	42.27	2	12.34	0 (0–10)	DF-5.5%	7.09
		(±9.89)	(0-6)	(±7.44)		Te-26.5%	(±8.738)
						IFNb-12.5%	
						GA-25%	
						F-11.5%	
						A-12.5%	
						N-6.5%	

*DF, dimethyl fumarate; Te, teriflunomide; IFNb, interferon beta 1a; GA, glatiramer acetate; F, fingolimod; A, alemtuzumab; N, natalizumab*.

### MR Imaging Acquisition

MR imaging was performed on a 3 T GE Discovery 750w MR Scanner (GE Healthcare, Chalfont St. Giles, UK). In total, MR images from 79 relapsing-remitting MS patients were acquired as part of the routine clinical follow-up based on our recent recommendation (17). In the analysis, the following sequences were used: high-resolution T1 weighted anatomical images [3D spoiled gradient-echo images with inversion recovery 3D FSPGR IR: echo time (TE): 5.4 ms; repetition time (TR): 2 ms; inversion time: 450 ms; matrix: 256^*^256; field of view (FOV): 25.6 × 25.6 cm; flip angle:12 degree; slice thickness: 1 mm; PURE intensity correction, NEX:1], CUBE T2 FLAIR for lesion detection (TE: 135 ms; TR: 6,700 ms; TI: 1,827 ms; matrix: 256 × 224; FOV: 25 × 22.5 cm; slice thickness: 1.4 mm; post-processing: ZIP512, ZIP2, NEX:1), SE T1 weighted images (TE: min full—minimum TE without fractional TE; TR: 500 ms; flip angle: 73 degree; matrix: 256 × 224; FOV: 24 × 19.2 cm; slice thickness: 3 mm; NEX:2).

### Image Analysis

All the image analyses were carried out using the tools of the FSL software package (https://fsl.fmrib.ox.ac.uk/fsl/fslwiki/FSL). All image (SE, FSPGR, and FLAIR) sequences were intensity normalized prior to the following steps (18). After removing the non-brain parts using BET (19), manual lesion-outlining on FLAIR images for all patients was performed by KK and supervised by KZT. In order to analyze only those lesions that are detectable on the 3 mm slice thickness images with certainty, lesions larger than 100 voxels were used in the analyses. SE and FLAIR images were registered to 3D FSPGR IR images with 6 DOF using FLIRT (20). The manually outlined and lesion masks were brought into FSPGR space using the previous registration matrices and trilinear interpolation. Masks were thresholded at 0.5 and binarized to avoid the enlargement due to the interpolation. Intensity values under the individual lesion masks on both FSPGR and SE images were extracted.

Tissue-type segmentation of the 3D FSPGR IR images was performed by FMRIB's Automated Segmentation Tool (FAST) (21). Lesions were masked out from the white matter partial volume images. Median intensity of the white matter, gray matter, and cerebrospinal fluid (CSF) was defined on each sequence. In order to estimate normalized brain volumes from FSPGR IR images, FSL SIENAX was used (22).

### Statistical Analysis

Altogether, data from 694 lesions was used in the statistical analyses. The lesions' median intensity values were calculated on FSPGR and SE sequences and were divided by the median intensity values of the white matter. These standardized intensities of the FSPGR and SE sequences were analyzed with one-sample Wilcoxon signed rank test to test whether the lesions can be differentiated from the surrounding white matter (a ratio different from 1).

In order to separate two different lesion types, K-means clustering was carried out (IBM SPSS Statistic 23, https://www.ibm.com/products/spss-statistics) on the lesion's median intensity values. Median lesion intensities per lesion from the FSPGR, SE, and FLAIR sequences were used, the number of clusters was set to 2; the iterate and classify method was used, and the number of maximum iterations was set to 10. The K-means cluster algorithm creates clusters from the dataset, placing centroids in a way that the data in a given cluster have similar attributes or closeness to the centroid, whilst the distance between clusters (centroids) is maximized (23). In order to quantify how median intensity values of the two lesion type clusters differ from the normal white matter intensity profile, we employed a bootstrap-based approach using a custom-made MATLAB script. To build up a null distribution for each subject, 5,000 random intensity values were sampled from subjectwise white matter voxels. A mean distance, defined as the average ratio of all white matter voxel intensities and the reference voxel intensity was calculated for all 5,000 selected voxels. The similar distance of lesions' median intensity was compared to this null-distribution and converted to Z-scores (which we refer to later as standardized lesion distance). This way, Z-scores take individual white matter intensity profiles into account. To assess how lesion type clusters can be separated from white matter voxels, we calculated the percentage of lesions exceeding a standardized lesion distance of *Z* = 2.3 for both lesion type clusters in both sequences.

Bivariate Spearman rank correlation between clinical parameters (EDSS, disease duration, number of relapses, and number of relapses since the beginning of the treatment), normalized brain volumes, and the number and volume of lesions in the clusters was calculated. All results were corrected for multiple comparison.

## Results

### Separability of Lesions From the Surrounding White Matter on T1 Weighted Images

Our results showed that lesions on average were separable from the surrounding white matter on both FSPGR and SE images. The intensity of the lesions was lower than white matter but higher than gray matter (and CSF) (FSPGR CSF: *Z* = 22.82, *p* ≤ 0.001; SE CSF: *Z* = 22.8, *p* ≤ 0.001; FSPGR GM: *Z* = 13.83, *p* ≤ 0.001; SE GM: *Z* = 21.34, *p* ≤ 0.001; FSPGR WM: *Z* = −22.81, *p* ≤ 0.001; SE WM: *Z* = −19.1, *p* ≤ 0.001). The standardized intensity of lesions on the SE and FSPGR images were different (normalized to white matter: *U* = 76491.5, *p* ≤ 0.001).

**Figure 1:**
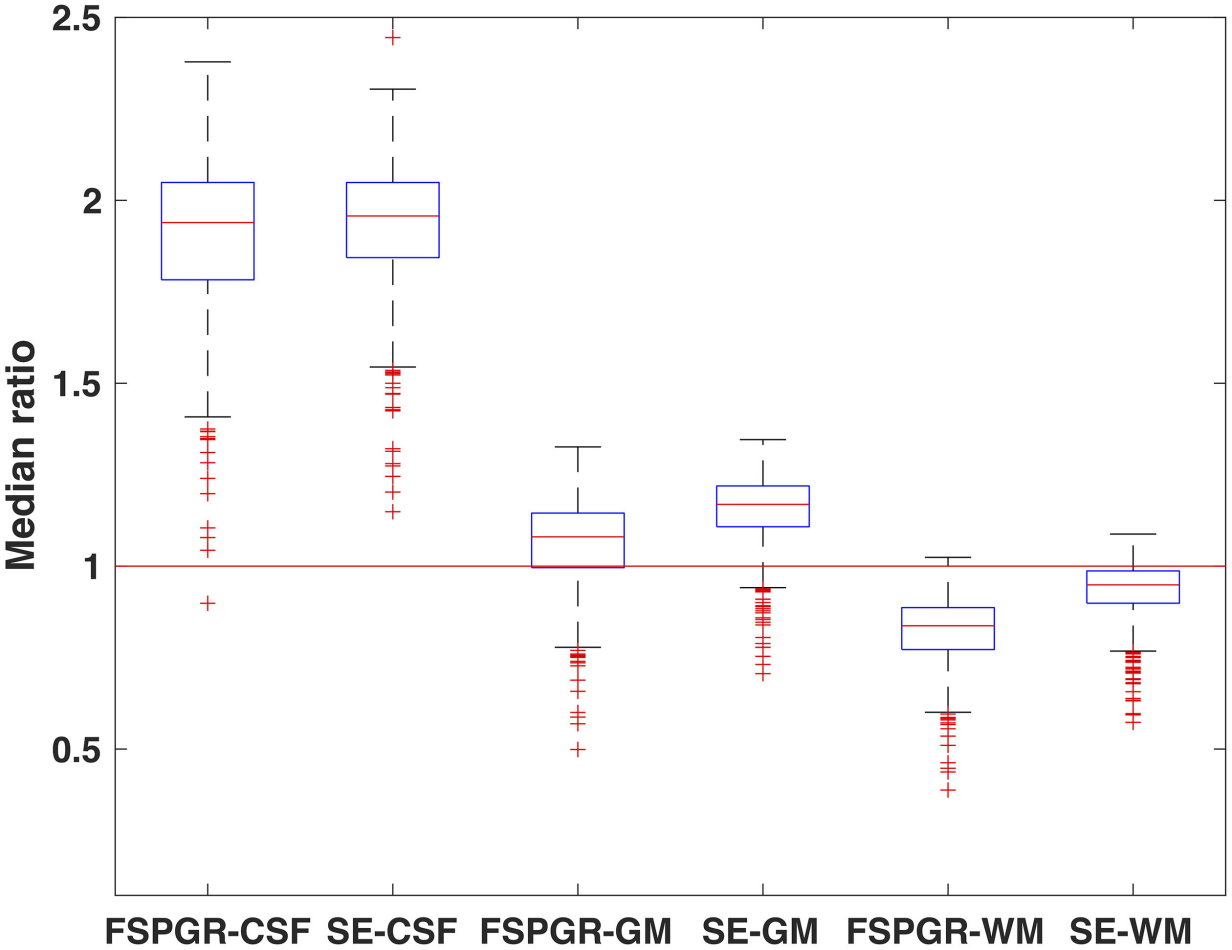
Standardized intensities of lesions on FSPGR and SE images. The median intensities of lesions were divided by the median intensity of the CSF, GM, and WM, respectively. Hence, a standardized intensity value of 1 would mean lesion intensity that does not stand out from the surrounding tissue intensity.

Albeit the intensity distributions of white matter lesions on both the SE and FSPGR images partly overlapped with the white matter intensities, the overlapping of the lesions on SE images was more expressed (Figure 1).

### Grouping the Lesions Based on Their Intensity

**Figure 2:**
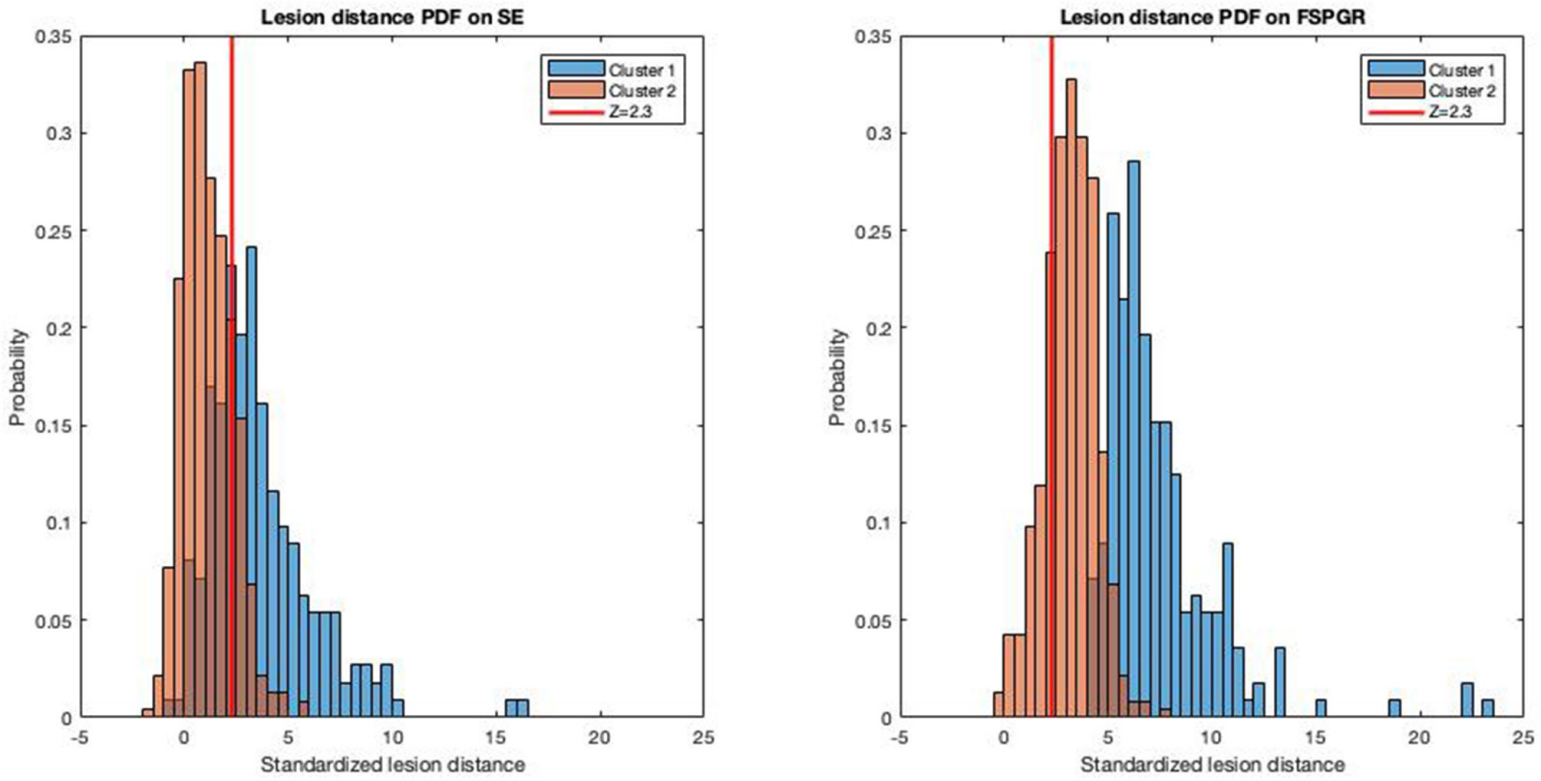
Separability of lesion clusters from white matter intensity in FSPGR and SE sequences. Histograms depict the probability density function of standardized lesion distance (see Methods for more details) for the two lesion type clusters on FSPGR and SE sequences. The vertical red line depicts a standardized lesion distance of *Z* = 2.3. PDF, probability density function.

**Figure 3:**
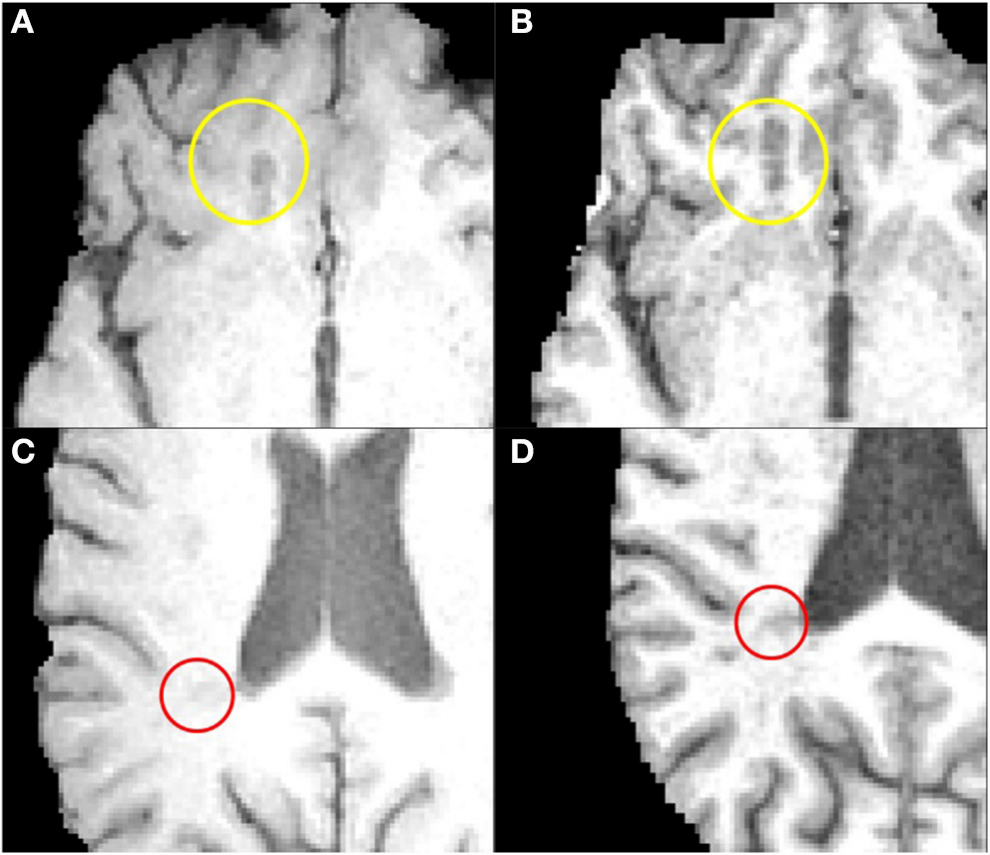
The two clusters of lesions on FSPGR and SE images. Images on the left **(A,C)** are examples depict SE whilst images on the right **(B,D)** depict FSPGR for Cluster 1 (yellow circle) and Cluster 2 (red circle), respectively.

Based on their intensity values on the FSPGR, SE, and FLAIR sequences, the K-means clustering algorithm grouped the 694 lesions into two clusters: 224 in Cluster 1 and 470 lesions in Cluster 2 (Figures 2, 3). The median intensity of lesions on FSPGR sequence had the maximum influence in the formation of the clusters (*F* = 1145.97), whilst median intensity values of lesions on FLAIR had the most negligible effect on it (*F* = 0.74). The main characteristics of the two clusters of lesions is summarized in Table 2.

**Table 2:** Summary of the results of K-means clustering.

	**1st cluster**	**2nd cluster**
Number of cases	*n* = 224	*n* = 470
Final cluster centers on FSPGR (intensity value)	3525.20	4280.42
Final cluster centers on SE (intensity value)	1701.23	1887.77
Final cluster centers on FLAIR (intensity value)	2575.23	2555.67
Median intensity (range) on FSPGR images	3,561 (2,259–3,886)	4,233 (3,988–4,797)
Median intensity (range) on SE images	1,732 (1,142–2,022)	1,902 (1,717–2,090)
Median intensity (range) on FLAIR images	2,558 (1,757–3,048)	2,603 (1,726–3,116)

*The table shows the main characteristics of the two clusters of lesions derived from K-means clustering*.

The percentage of Cluster 1 lesions exceeded a standardized lesion distance of *Z* = 2.3 was 100% for the FSPGR-sequence and 69% for the SE-sequence. As for Cluster 2 lesions, 78.7% exceeded a standardized lesion distance of *Z* = 2.3 on the FSPGR-sequence and 17.7% on the SE-sequence (see Figure 2 for further details). In summary, lesions in the second cluster on FSPGR images were clearly separable from the surrounding white matter; however, on SE images, the lesions were close to the mean intensity of the white matter, hence harder to visually detect.

### Clinical Relevance of the Lesion Types

Spearman rank correlation values are summarized in Table 3. Our results showed that the lesion count in Cluster 2 correlated significantly with EDSS (*R* = 0.3, *p* ≤ 0.042), with disease duration; (*R* = 0.33, *p* ≤ 0.014), and with the normalized brain volume (*R* = −0.42, *p* ≤ 0.008). Also, the lesion volume of Cluster 2 showed significant correlation with EDSS (*R* = 0.28, *p* ≤ 0.07) disease duration (*R* = 0.41, *p* ≤ 0.007), and with the normalized brain volume (*R* = −0.51, *p* ≤ 0.008), whilst no significant correlation was found with lesion volume and lesion count of the lesions in the Cluster 1.

**Table 3:** Significant correlations between clinical data and the volume and count of clustered lesions.

	**EDSS**	**Disease duration**	**Number of relapses**	**Number of relapses since treatment onset**	**Normalized brain volume**
CL-1 lesion count	*R* = 0.12, *p* = 1.0	*R* = 0.05, *p* = 1.0	*R* = 0.006, *p* = 1.0	*R* = −0.15, *p* = 1.0	*R* = −0.06, *p* = 1.0
CL-1 lesion volume	*R* = 0.14, *p* = 1.0	*R* = 0.008, *p* = 1.0	*R* = 0.07, *p* = 1.0	*R* = −0.18, *p* = 0.77	*R* = −0.09, *p* = 1.0
Cl-2 lesion count	***R*** **=** **0.30**, ***p*** **=** **0.042**	***R*** **=** **0.33**, ***p*** **=** **0.014**	*R* = 0.02, *p* = 1.0	*R* = 0.13, *p* = 1.0	***R*** **=** **–0.42**, ***p*** **=** **0.008**
CL-2 lesion volume	***R*** **=** **0.28**, ***p*** **=** **0.007**	***R*** **=** **0.41**, ***p*** **=** **0.007**	*R* = 0.18, *p* = 0.70	*R* = 0.28, *p* = 0.07	***R*** **=** **–0.51**, ***p*** **=** **0.008**
Lesion volume (total)	***R*** **=** **0.46**, ***p*** **=** **0.007**	***R*** **=** **0.33**, ***p*** **=** **0.014**	*R* = 0.21, *p* = 0.36	*R* = 0.21, *p* = 0.38	–
Lesion count (total)	*R* = 0.29, *p* = 0.056	*R* = 0.28, *p* = 0.07	*R* = −0.002, *p* = 1.0	*R* = 0.04, *p* = 1.0	–
Normalized brain volume	***R*** **=** **–0.46**, ***p*** **=** **0.008**	***R*** **=–0.38**, ***p*** **=** **0.008**	***R*** **=** **–0.36**, ***p*** **=** **0.008**	***R*** **=** **–0.31**, ***p*** **=** **0.036**	–

*Table 2 also shows significant correlations between normalized brain volume and clinical data and the volume and count of clustered lesions (CL-1, 1st Cluster; CL-2, 2nd cluster, EDSS, Expanded Disability Status Scale). The bold values highlight the significant correlations*.

Total lesion volume showed significant correlation with the EDSS scores (*R* = 0.46, *p* ≤ 0.007), and disease duration (*R* = 0.33, *p* ≤ 0.014).

**Figure 4:**
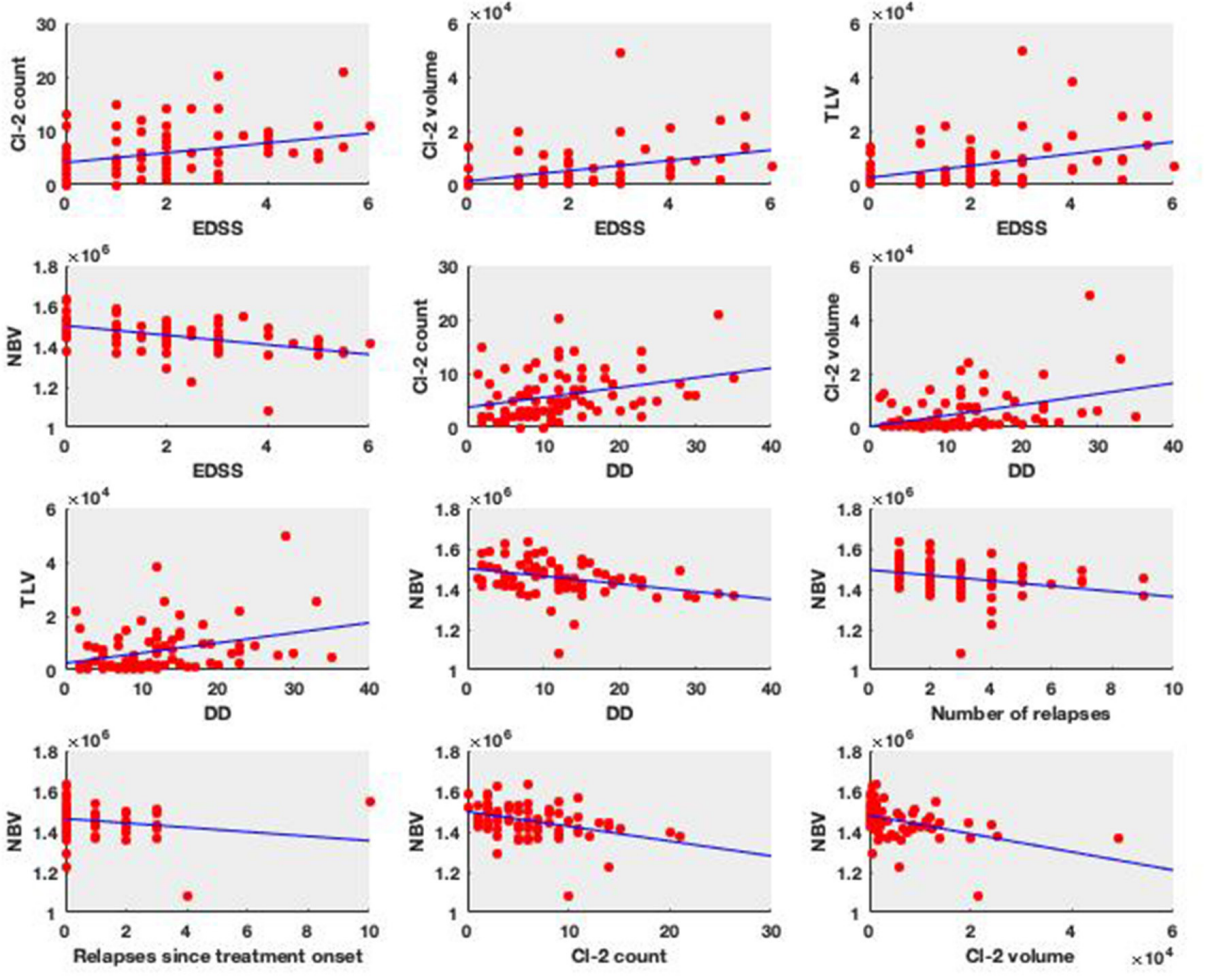
Clinical relevance of the lesion types. The subplots show the significant Spearman correlation plots (EDSS, Expanded Disability Status Scale; DD, disease duration; Cl-2 count, lesion count in the Cluster #2; Cl-2 volume, lesion volume in the Cluster #2; TLV, total lesion volume; NBV, normalized brain volume).

Furthermore, significant correlation was found between the normalized brain volumes and EDSS scores (*R* = −0.46, *p* ≤ 0.008), disease duration (*R* = −0.38, *p* ≤ 0.008), number of relapses (*R* = −0.36, *p* ≤ 0.008), and the number of relapses since treatment onset (*R* = −0.31, *p* ≤ 0.036). Scatterplots depicting the significant correlations are summarized in Figure 4.

## Discussion

In this study, we showed that (i) T1 hypointense lesions are more easily detectable using an FSPGR sequence than an SE sequence; (ii) Almost all of the T2 hyperintense lesions were easily detectable on FSPGR, but only less than half of the lesions were easily detected using a SE sequence; (iii) Interestingly, lesions not standing out on SE sequences are the one which are clinically more important; and (iv) the very same lesions are correlated with brain atrophy.

Our results are interesting in the view of earlier investigations suggesting a stronger correlation between clinical variables and SE T1 hypointense lesions than T2 hyperintense lesions. Truyen and colleagues showed that hypointense lesion load on the SE images correlated with EDSS, but T2 hyperintense lesion load did not. Importantly, this strong correlation was only significant for secondary progressive MS (SPMS) patients (5). Enzinger presented univariate correlation between the black-hole ratio on SE (personal communication) at baseline and MS severity score 10 years later, but the association did not hold if multiple regression model was used (24). The baseline black-hole ratio was a significant predictor of conversion to secondary-progressive multiple sclerosis (SPMS), but when baseline clinical variables were inserted into the models no significant predictive value remained among the investigated MRI parameters (24). In a Multiple Sclerosis Collaborative Research Group (MSCRG) trial of interferon beta-1a, there was a significant correlation between baseline T1 lesion load (presumably SE) and EDSS, but the correlation with T2 lesion load was virtually identical (25). Similarly, in Giorgio's study, both the volume and count of baseline T1w and T2w lesions correlated with the EDSS scores, but the combination of baseline count and volume of T1w lesions predicted more precisely the 10-year EDSS worsening (8). Moreover, most of the studies describing correlation between clinical parameters and black-hole lesion load were carried out on SPMS patients (26, 27).

We showed that the volume and number of T1 hypointense lesions in Cluster 2 significantly correlated with the normalized brain volumes of the patients. There are only a few publications examining the relationship between BHs and cerebral atrophy. Sailer et al. showed that an increase in T1 lesion load correlated with the increase of cerebral atrophy during an 18-month follow-up study (28). Paolillo et al. found significant correlation between the supratentorial brain volume and T1 total hypointense lesion load (11). Both studies suggested that the link between the volume increase of T1 hypointense lesions and cerebral atrophy is that local tissue damages and axonal loss might lead to increased neuronal packing density as well as contraction scarring caused by gliosis.

Our results are concordant with these former studies in that there was no correlation between Cluster 1 lesion number or volume with the clinical parameters. These are the lesions that were well differentiated on SE sequence from surrounding white matter, most comparable to traditional black-holes detected on SE images.

The importance of our findings is emphasized by Tam's studies (16, 29). The first study of a smaller population of mixed RR and SPMS patients showed stronger correlation between EDSS and black-hole volume when only the darkest voxels were considered (16). The consecutive study in a larger SPMS group showed the opposite, best correlation between MSFC when the brightest part of the hypointense lesions were also considered (most inclusive lesion masks) (29). These results were strengthened by incorporating T1 relaxation times in the analysis (3). While these studies only included SE images (personal communication), the importance of intensity variation of T1 hypointense lesions is clear. Along those lines, Adusumilli used a weighted lesion burden in which intensity values closer to CSF contribute most to the T1 hypointense lesion burden (15). Their measure shows good correlation with clinical and cognitive functions of the patients.

The importance of the intensity variation in the BHs is also supported by the histopathological studies. The degree of hypointensity correlated strongly with the axonal density in the lesions but not with the degree of demyelination or the number of reactive astrocytes (10). Fisher found that the contrast ratio of the T1 and MTR images (lesion intensity compared to the mean NAWM intensity) highly correlated with the axon count (9). The contrast ratios (T1, MTR, and FLAIR) differed significantly between myelinated and demyelinated lesions, but in the demyelinated lesions, the axon count correlated with the T1 and MTR contrast ratio, but not with the FLAIR contrast ratio. Importantly, chronic inactive lesions had the lowest contrast ratio (darkest lesions) as compared to active and chronic active lesions.

The question arises: Why did Cluster 2, with the relatively lighter lesions, show correlation with clinical measures, if the most severe tissue destruction is at the darkest part of the hypointense lesions? According to Fisher's results, the most hypointense part of the lesions are the inactive chronic lesions (9). One might speculate that these lesions are the oldest; hence, the most time was available for compensatory mechanisms and neural plasticity.

Here we used an automatic clustering approach to identify different types of lesions based on median intensity values on commonly used sequences. There were already studies to classify lesions, but some of those used images not included in the standard clinical routine (e.g., DTI, MTR, relaxometry) (30), or did not use a model-free approach (31).

Our work is not without limitations. Acquiring SE and FSPGR images with a set of different parameters would strengthen our results. The different spatial resolution of the two sequences might introduce a bias, but using these sequences we were investigating a real-life problem. Investigating various patient populations (RRMS and SPMS) and including patients with contrast-enhancing active lesion could further elaborate our findings.

## Conclusion

Our results call attention to consider FSPGR and SE sequences together when evaluating BHs. The clinical relevance of the intensity variation of the hypointense lesion is critical. Further studies are warranted to find a standardization approach and cutoff values for the FSPGR images that could result in reducing the number of measurements (e.g., skipping the SE sequence).

## Data Availability Statement

The raw data supporting the conclusions of this article will be made available by the authors, without undue reservation.

## Ethics Statement

The studies involving human participants were reviewed and approved by Regional Human Medical Ethics Committee University of Szeged, Szeged, Hungary. The patients/participants provided their written informed consent to participate in this study.

## Author Contributions

All authors listed have made a substantial, direct and intellectual contribution to the work, and approved it for publication.

## Conflict of Interest

The authors declare that the research was conducted in the absence of any commercial or financial relationships that could be construed as a potential conflict of interest.
